# Development of Antimicrobial Chitosan-Silver and Chitosan-Gold Nanocomposite Films by 3D Printing for Skin Application

**DOI:** 10.1021/acsomega.6c07342

**Published:** 2026-09-11

**Authors:** Rita de Cássia dos Reis Schmidt, Vitória Espinosa de Oliveira, Tainá Griebler, Júlia Lazzarini Machado, Leonardo Pessoa da Silva, Henrique Trombini, Marcelo Barbalho Pereira, Cesar Liberato Petzhold, Gabrielle Aguiar Dantas, Roberto Christ Vianna Santos, Nadine Lysyk Funk, Ruy Carlos Ruver Beck, Karina Paese, Monique Deon

**Affiliations:** † 117303 Universidade Federal de Ciências da Saúde de Porto Alegre, Programa de Pós-Graduação em Biociências, Rua Sarmento Leite 245, Porto Alegre, Rio Grande do Sul CEP 90050-170, Brazil; ‡ Universidade Federal de Ciências da Saúde de Porto Alegre, Rua Sarmento Leite 245, Porto Alegre, Rio Grande do Sul CEP 90050-170, Brazil; § 28124 Universidade Federal do Rio Grande do Sul, Instituto de Física, CP 15051, Porto Alegre, Rio Grande do Sul CEP 91501-970, Brazil; ∥ Universidade Federal do Rio Grande do Sul, Instituto de Química, CP 15003, Porto Alegre, Rio Grande do Sul CEP 91501-970, Brazil; ⊥ Laboratory of Oral Microbiology Research, 28118 Universidade Federal de Santa Maria, Av. Roraima 1000, Santa Maria, Rio Grande do Sul CEP 97105-900, Brazil; # Universidade Federal do Rio Grande do Sul, Programa de Pós-Graduação em Ciências Farmacêuticas, Av. Ipiranga 2752, Porto Alegre, Rio Grande do Sul CEP 90610-000, Brazil

## Abstract

Silver (AgNP) and gold (AuNP) nanoparticles are notable in nanomedicine for their unique properties and strong antibacterial effects; however, formulating them into skin-applied systems that preserve bioactivity while controlling nanoparticle exposure remains challenging. Herein, semisolid extrusion 3D printing of chitosan nanocomposite hydrogels loaded with AgNPs or AuNPs was used to manufacture personalized topical films with antimicrobial performance and minimized skin penetration risk. AgNPs and AuNPs were synthesized with comparable nanoscale dimensions (11.83 ± 3.74 nm and 11.67 ± 1.96 nm), enabling their uniform incorporation into chitosan matrices. The resulting inks presented topical-compatible pH (>4.0) and pronounced thixotropy with >90% viscosity recovery, translating into adequate performance in 3D printing. Nanoparticle incorporation afforded the tuning of properties such as swelling, mechanical behavior, and bioadhesion. Functionally, AgNP-loaded films exhibited the strongest broad-spectrum activity against *Staphylococcus aureus*, *Pseudomonas aeruginosa*, and *Candida albicans*. *Ex vivo* porcine skin studies indicated that free nanoparticles were detectable only in the stratum corneum, whereas metals were not detected after application of the 3D-printed films, supporting effective nanoparticle confinement by the chitosan matrix. These results position 3D-printed chitosan-nanoparticle films as a promising platform for customizable antimicrobial dressings for infected skin wounds.

## Graphical abstract

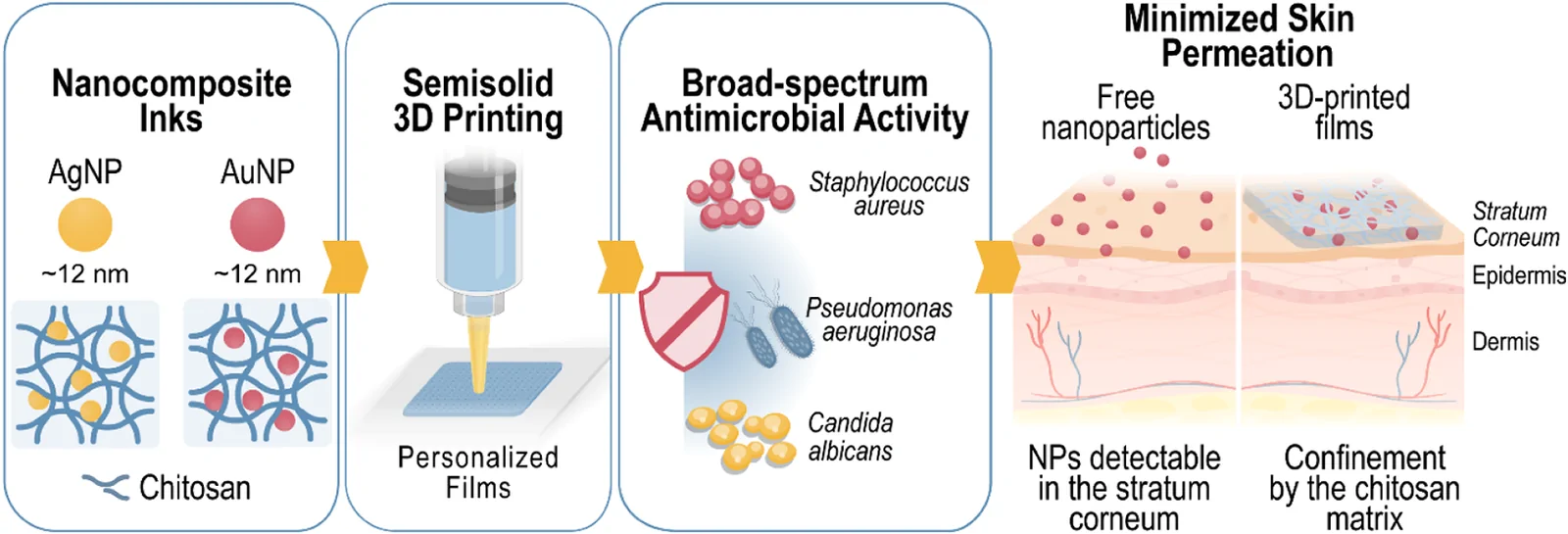

## 1 Introduction

The skin is the largest organ of the human body, acting as a protective barrier against pathogens, chemicals, and physical aggression, as well as regulating temperature and preventing dehydration.^1^ Damage or injury to the skin can result in wounds, for which effective treatments include the use of well-designed dressings, ensuring healing, protection, and comfort.^2,3^ Dressings should stop bleeding, promote hemostasis, regulate the moisture and oxygenation of the wound by absorbing exudates, and prevent microbial infections.^4^ With the rise of antimicrobial resistance among certain microorganisms, the treatment of infections in skin wounds has become more complex, requiring broad-spectrum antimicrobial agents to prevent microbial proliferation and promote proper healing.^3^

In this scenario, nanotechnology emerges as a promising strategy for the treatment and control of infections.^5^ Metal-based nanoparticles, such as silver (AgNPs), gold (AuNPs), copper, zinc oxide, and copper oxide, have been widely studied for their antimicrobial properties.^6^ These nanoparticles exhibit unique chemical, physical, and biological properties that are dependent on their size and shape, granting them tunable characteristics with high potential as emerging nanoantibiotics.^7,8^ Among these materials, AgNPs are particularly recognized for their potent and broad-spectrum antimicrobial activity, including against multidrug-resistant pathogens. Silver nanomaterials are also widely represented in commercially available nanomaterial-based products across sectors, such as textiles, cosmetics, and medical products.^9^ However, concerns regarding the cytotoxicity of AgNPs have encouraged further investigations of AuNPs, which may exhibit antimicrobial activity while presenting high colloidal stability, controllable size, and, most importantly, a favorable safety profile.^10^ The antimicrobial activity of AgNPs and AuNPs against multidrug-resistant pathogens can be attributed to their multiple mechanisms of action. These mechanisms include disrupting microbial membrane integrity, interacting with intracellular proteins and DNA, inducing oxidative stress, and inhibiting essential metabolic pathways.^11,12^ By targeting various cellular structures and functions simultaneously, these nanoparticles minimize the likelihood of bacterial resistance developing.^7,8,10^ In the treatment of infected skin wounds, the broad-spectrum antimicrobial activity of AgNPs and AuNPs has been shown against clinically relevant multidrug-resistant and biofilm-forming pathogens,^13^ including *Staphylococcus aureus*,^14,15^ *Pseudomonas aeruginosa*,^16^ and *Candida albicans*,^17^ which are among the most common microorganisms associated with chronic wound infections.

Despite their potential, the biological application of metal nanoparticles still faces challenges, such as low colloidal stability in their free form.^18^ It is important to understand how nanoparticles interact with biological environments, especially for skin applications. Consequently, numerous studies have been conducted to investigate the penetration and bioaccumulation of AgNPs and AuNPs, to determine their suitability for topical use.^19,20^ The absorption and penetration of these nanoparticles can vary based on their size, surface properties, and specific area of the skin where they are applied.^20^ Skin penetration and permeation studies have demonstrated that nanoparticles can be absorbed through intercellular and follicular pathways,^21^ although the exact mechanisms still need further elucidation.

To address these challenges, the incorporation of metal nanoparticles into polymeric matrices has become increasingly important. This approach not only enhances the colloidal stability of the nanoparticles but also allows for the creation of polymeric nanocomposites, keeping the particles confined within the matrix and improving their stability and applicability.^13^ The inherent properties of polymers, such as mechanical strength, fluid absorption (especially in hydrophilic polymers), biocompatibility, and flexibility, play a significant role in determining their effectiveness.^22^ When combined with the antimicrobial action of inorganic nanoparticles, polymeric nanocomposites show great potential for use in smart dressings. Their polymeric matrix confines nanoparticles, enabling localized antimicrobial activity while minimizing direct release into the skin, which reduces cytotoxic effects and enhances clinical applicability.^23^

Chitosan is a natural polysaccharide derived from chitin, which is widely available and extracted from crustacean shells.^24^ Chitosan has gathered attention in the biomedical field for its nontoxic, biocompatible, biodegradable, moisture-retaining, and cost-effective properties.^25^ Recent studies emphasize its significant potential for skin applications and tissue engineering due to its capacity to enhance cell regeneration, particularly in wound dressings.^26^ Chitosan has inherent antibacterial properties due to amino groups that, when protonated, interact with the bacterial cell wall. However, this activity can decrease at neutral or basic pH levels,^25^ and the combination of chitosan with metal nanoparticles is a strategy for enhancing its antimicrobial effectiveness.^27–30^ Additionally, as a hydrophilic polymer, chitosan demonstrates a strong capacity to form hydrogels in acidic environments, facilitating the uniform incorporation of nanoparticles into its matrix.^31^

To develop customized polymeric nanocomposites for wound dressings, 3D printing has emerged as a versatile technology in biomaterial engineering. Among the available 3D printing techniques, semisolid extrusion (SSE) enables the use of hydrogel formulations as inks for printing because it processes viscous, non-thermoplastic materials under relatively mild conditions, which could be modified with nanomaterials such as AuNPs and AgNPs.^32,33^ The success of this technique relies on the rheological and structural properties of the nanocomposite ink, which are essential for ensuring adequate flow during extrusion and rapid structural recovery after deposition, which is required to ensure printing accuracy and shape retention of the final product. Compared with conventional solvent casting, SSE provides layer-by-layer control over the film geometry, surface area, and thickness. This enables the fabrication of devices with dimensions adapted to the wound and allows for control over the amount of nanocomposite deposited in each film. In an on-demand manufacturing context, SSE may also reduce material waste because films can be printed directly in the required geometry, whereas cast films are typically produced as standardized sheets and subsequently cut to size.^34–36^ These features make SSE a relevant platform for manufacturing chitosan-based films containing AgNPs or AuNPs, particularly when customization of device dimensions and composition is desired.

Recent studies have demonstrated the potential of 3D-printed hydrogel systems containing metal nanoparticles for wound-related applications, particularly those incorporating AgNPs.^34,37^ AgNP-containing printed hydrogels based on alginate,^38,39^ GelMA,^40^ and modified chitosan matrices have shown antimicrobial activity and,^39,41^ in some cases, improved wound closure, re-epithelialization, and tissue regeneration. In contrast, although AuNP-containing hydrogels have been investigated for wound healing,^42,43^ their use in 3D-printed skin or wound systems is scarcely explored.^44^ Moreover, comparisons between AgNPs and AuNPs within the same polymer matrix remain limited^45^ and have not been applied to printable formulations. This gap is particularly relevant for chitosan-based semisolid extrusion systems, in which nanoparticle incorporation may influence rheological behavior and printability, as well as the physicochemical, mechanical, and biological properties of the printed material when intended for customizable wound dressings.

In this context, the present study reports the development and comparative evaluation of biopolymeric nanocomposite films based on chitosan, containing AgNPs and AuNPs, produced by the SSE 3D printing technique. The two nanocomposite systems were investigated under the same formulation and processing framework with respect to rheological behavior, printability, physicochemical and mechanical properties, bioadhesion, antimicrobial activity, and ex vivo skin interactions.

## 2 Experimental Section

### 2.1 Chemicals

Gold powder (<45 μm, 99.99%), glutaraldehyde solution (25% in H_2_O), and chitosan with low molecular weight (50–190 kDa; deacetylation degree ≥75%) were all purchased from Sigma-Aldrich. Silver nitrate (AgNO_3_) (>99.0%) and glycerol (>99.5%) were purchased from Synth. Acetic acid (glacial 99.8%), sodium borohydride (NaBH_4_, ≥98.0%), sodium hydroxide (NaOH, ≥97.0%), anhydrous monobasic sodium phosphate (≥98.0%), and anhydrous dibasic sodium phosphate (≥98.0%) were all purchased from Neon. Acetone UV/HPLC (99.9%) was purchased from Dinâmica. Ultrapure water (Millipore water purification system) was used to prepare solutions.

### 2.2 Silver and Gold Nanoparticles

#### 2.2.1 Synthesis

The chloroauric acid (HAuCl_4_) solution at 0.02 mol L^–1^ was prepared by dissolving gold powder in a minimum volume of aqua regia, then diluting in ultrapure water. The AgNO_3_ (0.02 mol L^–1^) and NaBH_4_ (0.02 mol L^–1^) solutions were prepared in ultrapure water.

The synthesis of AgNPs and AuNPs was based on the chemical reduction method, in which Ag^+^ is reduced to Ag^0^ and Au^3+^ to Au^0^, following a previously reported method.^46^ Initially, 75 mg of the stabilizing agent chitosan was dissolved in 10.0 mL of a 2% acetic acid solution while being stirred magnetically at room temperature. In the synthesis of AgNPs, 1.0 mL of AgNO_3_ was added, followed by 5.0 mL of NaBH_4_ solution. This process resulted in a color change to yellow. For the synthesis of AuNPs, 1.0 mL of HAuCl_4_ was added, followed by the same amount of NaBH_4_ solution.^46^ This addition led to a color change to red.

#### 2.2.2 Characterization

The surface plasmon resonance (SPR) of nanoparticles was analyzed by their UV–vis extinction spectra, acquired with the UV-1800 spectrophotometer (Shimadzu). The samples were diluted 1:1 in ultrapure water, placed into quartz cuvettes, and analyzed in the wavelength range of 200 to 800 nm.

The size and morphology of nanoparticles were analyzed using transmission electron microscopy (TEM) and scanning transmission electron microscopy (STEM). TEM images were acquired with the JEOL 1400 Flash, operating at 120 kV. The samples were prepared by depositing 30 μL of nanoparticle dispersion on a 200-mesh copper grid coated with a carbon film. STEM was performed on an Inspect F50 scanning electron microscope (FEI Company) at an acceleration voltage of 30 kV. The samples were diluted in a 1:9 ratio (nanoparticle:water), and 30 μL was deposited onto a 400-mesh copper grid coated with a carbon film. The average size was estimated by measuring 200 nanoparticles using ImageJ software.

The surface charge of the nanoparticles was analyzed using zeta potential at the Zetasizer Ultra (Zetasizer Nano ZS, Malvern Instruments). The nanoparticles were dispersed in a 1:100 dilution in ultrapure water, filtered through a 0.45 μm membrane, and analyzed using the bucket model DTS1070.

### 2.3 Hydrogel Nanocomposites

#### 2.3.1 Preparation

Three hydrogels were prepared: CS/AgNP, a 6% chitosan hydrogel containing AgNPs; CS/AuNP, a 6% chitosan hydrogel containing AuNPs; and CS, a nanoparticle-free 6% chitosan hydrogel (blank). The chitosan concentration was selected based on preliminary formulation and printing tests that indicated suitable gel extrudability and shape retention for SSE 3D printing. Initially, a portion of 600 mg, corresponding to 6% of chitosan (w/w), was dissolved in 6.2 mL of the NP dispersion (AgNPs to CS/AgNP, AuNPs to CS/AuNP, and ultrapure water to CS), with 800 μL of acetic acid. Then, 1.0 g of glycerol (10%) was added as a plasticizing agent, and 1.4 mL of NaOH (1.0 mol L^–1^) was added to adjust the pH and improve the rheological properties required for SSE 3D printing.^47^ The final nanocomposite hydrogels contained 0.775 μmol of Ag or Au per gram of hydrogel.

#### 2.3.2 Characterization

For pH evaluation, initially, 1.0 g of hydrogel was added to 10 mL of ultrapure water and stirred at room temperature for 1 h. After total dispersion, the pH was measured with a pH meter FiveEasy (Mettler Toledo) at room temperature.

The rheological analyses were conducted using a rheometer Ares G2 (TA Instruments) with a 25 mm parallel plate, a measurement gap of 1 mm, and analyzed at 25 °C. All samples were measured in triplicate.

To define the linear viscoelastic region (LVR), the oscillatory amplitude test was conducted with a stress sweep of 0.1–500% and a constant frequency of 1 Hz. The viscoelastic properties of the hydrogels, including storage modulus (G’), loss modulus (G’’), and tangent delta (tan δ), were obtained through an oscillatory frequency test with an angular frequency sweep of 0.1 to 600 rad/s. To determine the best-fit flow, a flow ramp test was conducted with a shear rate range of 0.4 to 1.5 s^–1^. The thixotropy tests of hydrogels were conducted through oscillations in five stages. Initially, a strain of 2.5% was applied for 60 s, followed by a strain of 250% for 60 s. Afterward, the process was repeated, alternating between a strain of 2.5% and 250% for 60 s at each stage. Data collection and analyses were performed using TRIOS software (TA Instruments).

### 2.4 3D-Printed Nanocomposite Films

#### 2.4.1 3D Printing of Hydrogel Nanocomposites

The nanocomposite films from CS, CS/AgNP, and CS/AuNP hydrogels were designed using PrusaSlicer software (Prusa Research) and printed using SSE in the BioEnder Termo bioprinter (BioEdTech). The printing parameters used were as follows: room temperature for extrusion, nozzle diameter of 0.41 mm, layer height of 0.20 mm, number of layers of 5, and dimensions of 20 mm (length) × 20 mm (width) × 1 mm (height). The printing speed was 5 mm s^–1^, selected based on preliminary trials aimed at obtaining continuous extrusion. The extrusion conditions were maintained unchanged throughout the experiments to allow direct comparison among the formulations.

#### 2.4.2 Characterization

After printing, the weight of the films (n = 10) was measured and they were dried at room temperature. After drying for 72 h at room temperature, the weight of the films (n = 10) was measured using an analytical balance with a precision of five decimal places. The dimensions of the films were measured using a digital caliper. The results are expressed with mean ± standard deviation (SD).

The SPR bands of CS/AgNP and CS/AuNP nanocomposite films were studied using a UV–vis–NIR spectrophotometer (Varian Cary 5000) to measure total transmittance in the wavelength range of 200 to 800 nm.

The techniques of particle-induced X-ray emission (PIXE) spectroscopy and *μ*-particle-induced X-ray emission (*μ*-PIXE) were used to identify the elements in the surfaces of 3D-printed nanocomposite films. The PIXE analysis was employed to determine the elemental composition of the films CS, CS/AgNP, and CS/AuNP. The scanning *μ-*PIXE technique was used to evaluate the heterogeneity of nanoparticle distribution on the surfaces of the CS/AgNP and CS/AuNP films. The measurements were taken using the 6 MV Tandetron accelerator. In the PIXE, the ion beam H^+^ at 2 MeV was used, with a pressure in the analysis chamber of 10^–6^ mbar (high vacuum) and a Si (Li) detector. The *μ-*PIXE system analysis utilized ion beam H^+^ at 2.2 MeV, a pressure of 10^–6^ mbar, and an SDD-type detector. The μ-beam scan was performed over an area of 1 mm × 1 mm, with a beam spot size smaller than 3 μm. For all analyses, the total charge associated with the beam current was recorded, and the spectra were normalized by this charge. In all cases, the NIST SRM 1577c standard was used for system energy calibration. The GUPIXWIN software was used to quantitatively analyze the data.

The surface morphology and composition of the films were analyzed by using scanning electron microscopy (SEM) with an Inspect F50 scanning electron microscope (FEI Company). Two different detectors were employed at an acceleration voltage of 10 kV: a secondary electron detector (SE) for assessing surface morphology and a backscattered electron detector (BSE) for analyzing the composition of the films. The samples were mounted on aluminum stubs using double-sided carbon conductive tape and were coated with 10 nm of carbon using a sputter coater ACE600 (Leica).

Thermogravimetric analysis (TGA) was performed using the TGA-50 instrument (Shimadzu) to study the thermal decomposition profile of CS, CS/AgNP, and CS/AuNP film nanocomposites. The samples were heated from room temperature up to 900 °C, at 20 °C min^–1^, in an air atmosphere.

The tensile strength of nanocomposite films (n = 3) was measured using the Texture Analyzer (TA.XT plus, Stable Micro Systems), a piece of equipment equipped with a load cell 5 kg and tensile grips. For the analysis, the nanocomposite films were printed in specific dimensions: 50 mm (length) × 10 mm (width) × 1 mm (height). The samples were vertically clamped between two tensile grips at a distance of 30 mm, and each film was stretched at a crosshead speed of 0.5 mm/s until it broke. The collected data were analyzed using the software Exponent (Stable Micro Systems).

Experiments were conducted to evaluate the behavior of the 3D-printed nanocomposite films in aqueous environments, including their swelling, relative wet mass, and dry mass loss, adapted from Gudimalla et al.^48^ For these experiments, samples (n = 3) measuring 10 mm × 10 mm were used. In the swelling studies, the initial weight (*W*_0_) was measured, and the film was immersed in 10 mL of ultrapure water, ensuring it remained completely submerged. The swollen films were removed at specific time intervals, the excess water was removed using absorbent paper, and the weight was recorded (*W*_1_). The swelling process was monitored for 48 h, and the swelling degree was calculated using the following equation:

1 $$Swelling\;\%=\frac{W_{1}-W_{0}}{W_{0}}\times 100$$

The behavior in the aqueous environment was evaluated, monitoring the relative mass of each film (n = 3) over 48 h, in a phosphate buffer at a concentration of 0.1 mol L^–1^, at three different pH values: 4.0, 5.8, and 8.5. These pH levels were selected to investigate film behavior across acidic, mildly acidic, and alkaline environments relevant to the range of conditions reported at the skin and wound interface.^49,50^ The relative mass was then calculated according to the equation below, where *W*_1_ is the wet mass of the film at each time point and *W*_0_ the initial weight.

2 $$Relative\;Mass\;\%=\frac{W_{1}}{W_{0}}\times 100$$

To evaluate the dry mass loss of nanocomposite films in water and buffered media at different pH values (4.0, 5.8, and 8.5), the samples were dried in an oven at 105 °C for 12 h. After measuring the initial dry weight (*W*_0_), the 3D-printed nanocomposite films were immersed in 40 mL of the respective medium. The samples were left under magnetic stirring at 25 °C for 24 h. Afterward, the samples were dried again in an oven at 105 °C for 12 h. The final weight (*W*_1_) was recorded, and dry mass loss was calculated using the following equation:

3 $$Dry\;mass\;loss\;\%=\frac{W_{0}-W_{1}}{W_{0}}\times 100$$

#### 2.4.3 Biological Characterization

The bioadhesive potential was evaluated according to Osmari et al.,^51^ using a tensile stress tester (TA.XTplus Texture Analyzer). Porcine ear skin was fixed onto the platform, and 38 μL of ultrapure water was added to its surface. Circular samples of 10 mm from each film (n = 3) were placed on the equipment probe, which remained in contact with the skin for 1 min under a force of 1 N. The probe was then withdrawn at a constant rate of 0.10 mm/s until complete detachment. The peak force (mN), displacement, and work of adhesion were recorded using the Texture Exponent software (Stable Micro Systems).

The microorganisms *Candida albicans* (ATCC24433), *Staphylococcus aureus* (ATCC25923), and *Pseudomonas aeruginosa* (PA01) were used as indicators of antimicrobial activity. The absorbances were adjusted in sterile saline solution (0.85% NaCl) to 0.5 on the McFarland scale (∼1.5 × 10^8^ CFU mL^–1^) using a spectrophotometer (O.D. 600). Prior to testing, the 3D-printed films, with dimensions of 20 × 20 mm (approximately 0.45 μmol of Ag or Au per film), were cleaned with 70% ethanol and washed twice with sterile distilled water. The 3D-printed films were divided into the following groups: negative control (Tryptic Soy Broth + film); positive control (Tryptic Soy Broth + Microorganism); CS (Tryptic Soy Broth + CS + Microorganism); CS/AuNP (Tryptic Soy Broth + CS/AuNP + Microorganism); and CS/AgNP (Tryptic Soy Broth + CS/AgNP + Microorganism).

The assays were performed in triplicate and repeated three times independently. The tubes were incubated at 36 ± 1 °C for 24 h. The number of viable microorganisms was determined by the plate count method, and the results were expressed as the logarithm of the viable microorganism count (mean ± standard error).

The skin penetration of AgNPs and AuNPs was studied in porcine skin with free NPs (dispersion of AuNPs and AgNPs) and 3D-printed nanocomposite films CS/AgNP and CS/AuNP. A pool of porcine ears was used, and the experiment was based on the assay of Detoni et al.^52^ The porcine skin was cut into round slices measuring 2.5 cm × 2.5 cm, with an average thickness of 1.43 ± 0.047 mm. These slices were used as a biological membrane in a manual Franz cell apparatus with an exposure area of 2.54 cm^2^. Six experiments were conducted in which the skins were treated with ultrapure water, AgNP, AuNP, CS film, CS/AgNP film, and CS/AuNP film. For the experiments with free NPs, AuNP and AgNP dispersions (215 μL, corresponding to 0.270 μmol of Au or Ag) were diluted in ultrapure water up to 645 μL. For the sample treated with water, 645 μL of ultrapure water was applied. The films (CS, CS/AgNP, and CS/AuNP) were designed and printed with a contact area of 2.54 cm^2^ and contained the same amount of nanoparticles as those applied freely to the skin (*ca.* 0.270 μmol of Au or Ag). In the assay using the films, an additional 430 μL of ultrapure water was applied to maintain a hydrated environment. Ultrapure water was used as the receptor medium while constantly being stirred in a water bath at 32 °C. The experiment was conducted under these conditions for 24 h. Afterward, the skin was fixed with a solution of glutaraldehyde and phosphate buffer (pH 7.4) in a 1:1 ratio for 1 week. After this, the skin samples were washed three times for 30 min in a phosphate solution (0.1 mol L^–1^, pH 7.4) and dehydrated through serial washing with varying water–acetone ratios: 7:3, 5:5, 3:7, 1:9 (repeated twice), and finally 0:10 (repeated twice). The samples immersed in acetone were dried using a critical point dryer CPD030 (Bal-Tec). Subsequently, the samples were coated with 10 nm of carbon using a sputter coater ACE600 (Leica). The penetration of nanoparticles in the skin was analyzed using an Inspect F50 scanning electron microscope (FEI Company) with three different detectors: secondary electron detector (SE), backscattered electrons (BSE), and energy-dispersive X-ray spectroscopy (EDS, X-Max, Oxford Instruments).

### 2.5 Statistical Methods

Data are presented as the mean ± standard deviation (SD). Statistical analyses were performed using one-way analysis of variance (ANOVA) followed by Tukey’s multiple-comparisons test in GraphPad Prism version 10. Differences were considered statistically significant when *p* < 0.05.

## 3 Results and Discussion

### 3.1 Silver and Gold Nanoparticle Characterization

AgNP and AuNP formation was first confirmed by UV–vis spectroscopy, as the characteristic color changes (colorless to yellow for AgNPs and yellow to red for AuNPs) are resultant of the localized surface plasmon resonance (SPR), which originates from the collective oscillation of surface conduction electrons under incident light.^53,54^ The UV–vis spectra presented the characteristic SPR band of each nanoparticle, observed at 402 nm for AgNPs and 525 nm for AuNPs (Figure S1).

The morphology of the nanoparticles was evaluated using STEM (Figure 1) and TEM (Figure S2). Both techniques revealed spherical shapes for the AgNPs and AuNPs, which aligned with previously reported results.^55,56^ The size of the nanoparticles was estimated through both TEM and STEM, with values from 9 to 12 nm for both AgNPs and AuNPs, as shown in Table 1.

**Figure 1:**
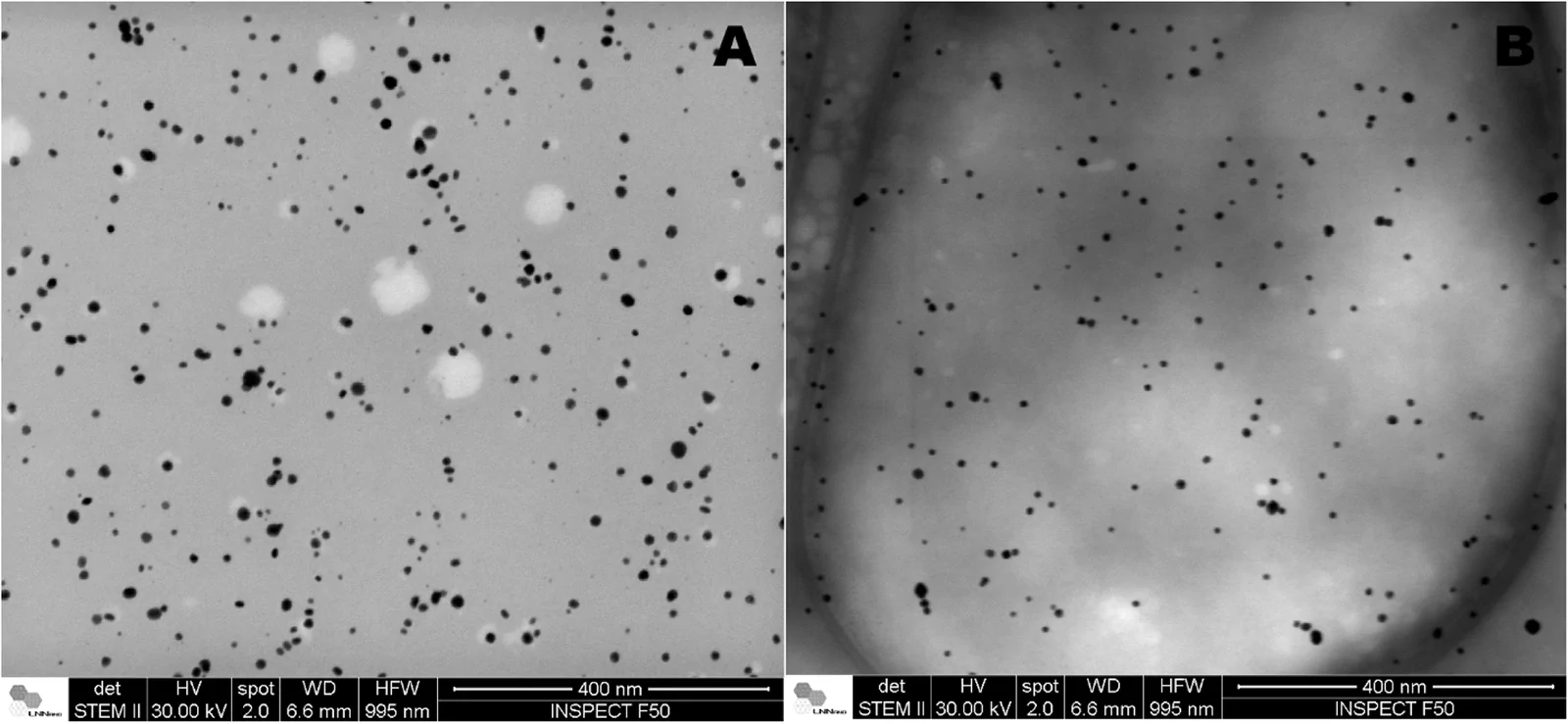
STEM images of (A) AgNPs and (B) AuNPs.

**Table 1:** Average Size and Zeta Potential of AgNPs and AuNPs

Nanoparticle	Size TEM (nm)^a^	Size STEM (nm)^a^	Zeta potential (mV)
AgNP	11.8 ± 3.7	12.4 ± 3.3	+56.6 ± 1.6
AuNP	11.7 ± 2.0	9.2 ± 2.5	+55.9 ± 2.1

a n = 200 nanoparticles.

Zeta potential analysis was used to determine the surface charge of nanoparticles, providing information about the stability of the system. The zeta potential is defined as the Helmholtz electric potential near the surface of a particle and the liquid when the particle is suspended.^57^ Particles with high zeta potential, whether negative or positive, tend to repel each other, which prevents their aggregation. The high zeta potential shown in Table 1 indicates good colloidal stability, as studies suggest that zeta potentials greater than ±30 mV correlate with enhanced stability in the system.^58^ Furthermore, the zeta potential of AgNPs and AuNPs stabilized with chitosan was reported to exceed +50 mV, consistent with findings from other studies.^59^ The high positive zeta potential arises from chitosan amino groups, which are protonated in acidic pH. This behavior allows the groups to repel each other, maintaining the stability and monodispersity of the nanoparticles.^60^

### 3.2 Hydrogel Nanocomposite Characterization

The pH of hydrogels was measured to investigate their potential effects on the skin, considering the pH of healthy skin and various types of wound healing including with infections. The pH of hydrogels was measured before and after adding NaOH, and the obtained values are presented in Table 2:

**Table 2:** pH and Rheological Characteristics of Hydrogel Nanocomposites

Hydrogel	pH before NaOH[Table-fn tbl2fn1] adding	pH after NaOH[Table-fn tbl2fn1] adding	Yield stress (Pa)	Complex viscosity η* at 1 Hz (Pa.S)
CS	3.66 ± 0.14	4.13 ± 0.05	258 ± 13^b^	619 ± 44^b^
CS/AgNP	3.80 ± 0.04	4.14 ± 0.03	285 ± 8^a^	625 ± 31^b^
CS/AuNP	3.80 ± 0.04	4.11 ± 0.03	265 ± 6^ab^	1830 ± 42^a^

i NaOH concentration = 1.0 mol L^–1^. Values are presented as mean ± SD (n = 3). Different superscript letters indicate statistically significant differences between groups within the same column (*p* < 0.05).

The addition of NaOH increased the pH of the hydrogels by partially deprotonating the −NH_3_^+^ groups of chitosan. These groups had previously been protonated in an acidic medium to solubilize chitosan. The addition of NaOH is reported to modify the rheological and mechanical properties of chitosan hydrogels while also increasing their crystallinity.^61^ These factors can enhance the printing quality of hydrogels. At the same time, the selected pH remained within the acidic range in which a substantial fraction of chitosan amino groups is expected to remain protonated. This is relevant because the intrinsic antimicrobial activity of chitosan is generally associated with its cationic character.^62^ Moreover, although the pH of the skin can vary by region, recent studies indicate that it typically ranges from 4.1 to 5.8, in alignment with the pH obtained for the hydrogel nanocomposites.^63^ This indicates that the formulations could be promising for skin applications, as demonstrated in previous works.^64–66^

Rheology plays an important role in 3D printing, therefore, it was assessed, and the corresponding results are presented in Figure 2. Understanding the rheological characteristics, such as shear thinning, yield stress, viscosity, fluidity, and thixotropy, is fundamental to ensure precise and controlled extrusion throughout the entire printing process.^67^ Additionally, incorporating fillers into the polymeric matrix to obtain nanocomposite hydrogels can alter the rheological properties, directly impacting their printability. These fillers can increase the viscosity of semisolid materials, which affects extrusion and printing quality, as noted in previous studies.^68,69^ This behavior is observed in Table 2, where the increase in complex viscosity (η*) of the CS/AuNP hydrogel nanocomposite becomes evident when compared with blank CS. In this context, AuNPs have been reported to interact with polymer chains,^70^ which can influence the rheological behavior of nanocomposites.

**Figure 2:**
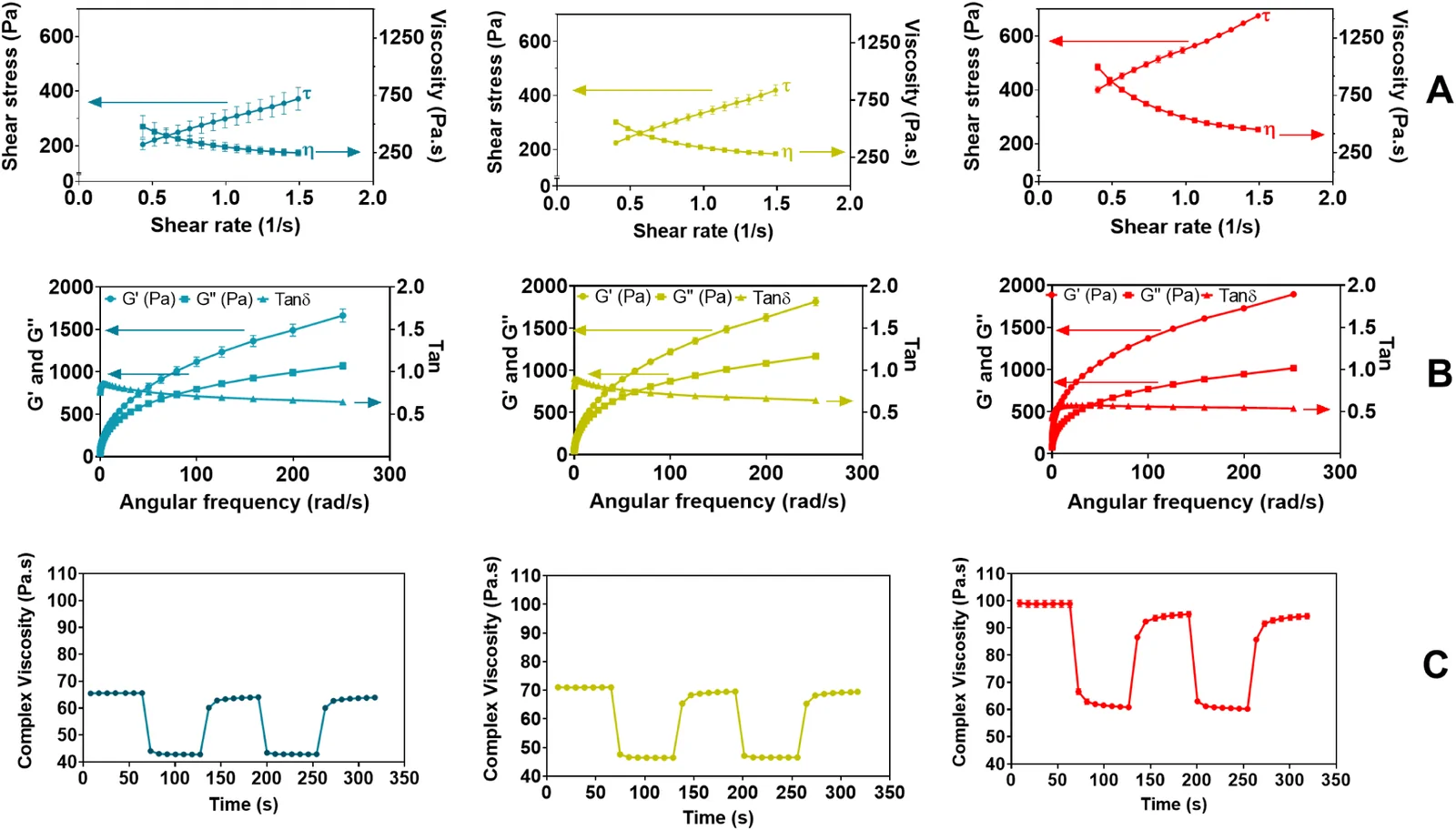
Rheology characterizations of hydrogels CS, CS/AgNP, and CS/AuNP. (A) Shear stress and viscosity versus shear rate, (B) storage modulus (G’), loss modulus (G”), and tangent delta (tan δ), and (C) thixotropy behavior depicted as time versus complex viscosity. Data are presented as mean ± standard deviation (n = 3).

Another important factor for SSE is yield stress, which is the minimum stress necessary for a material to begin flowing. The results are presented in Table 2. As shown in Figure 2, although CS/AuNP exhibits significantly higher complex viscosity, the yield stress results indicate similar yield stress for CS/AuNP when compared with CS/AgNP. Nevertheless, the results from the flow ramp tests indicate a decrease in apparent viscosity (η) with increasing shear stress (τ) for all samples (Figure 2). Therefore, both hydrogels (CS/AuNP and CS/AgNP) exhibited thinning behavior under shear, which is characteristic of materials classified as pseudoplastic.^68^ This behavior is ideal for materials intended for 3D printing by the SSE technique.

Semisolid materials, such as hydrogels, exhibit viscoelastic behavior. The key parameters used to characterize these materials are the storage modulus (G’), also known as the elastic modulus, and the loss modulus (G”), also referred to as the viscous modulus. Additionally, the ratio of these moduli is represented by the tangent delta (tan δ), which indicates the relationship between G’ and G’’. The storage modulus is related to the capacity of these materials to store and recover energy after the application of shear. Conversely, the loss modulus indicates the energy dissipated during deformation caused by applied shear.^68,71^ Figure 2B shows that the hydrogels CS, CS/AgNP, and CS/AuNP exhibited a higher storage modulus than the loss modulus, with tan δ values less than 1. This indicates that the hydrogels exhibit predominantly elastic behavior, with gel-like characteristics.^72,73^ This analysis suggests that these formulations may favor the retention of the object’s structure after printing.

These characteristics above can be confirmed through thixotropic analysis of hydrogels (Figure 2C). The hydrogels were submitted to five cycles of two different strains, 2.5% and 250%, during 60 seconds each. It was observed that after the high strain rate, the viscosities of CS, CS/AgNP, and CS/AuNP decreased. After the cessation of strain, the recovery of viscosity was measured as follows: 96.06% ± 0.04 for CS, 96.41% ± 0.06 for CS/AgNP, and 93.30% ± 0.24 for CS/AuNP. However, after 9 s of low strain, the recovery increased (see Figure 2C) to 96.82% ± 0.06 for CS, 97.15% ± 0.05 for CS/AgNP, and 94.40% ± 0.24 for CS/AuNP. These results demonstrate that the hydrogels are thixotropic and can restore their initial viscosity. These characteristics were fundamental to 3D printing for SSE, as they guarantee the fluidity necessary during extrusion and form recovery after deposition.^74^

### 3.3 3D-Printed Film Characterization

Firstly, the shape and size of the films were planned in the CAD (computer-aided design) model design process using PrusaSlicer software. Figure 3 shows the appearance of 3D-printed films from CS, CS/AgNP, and CS/AuNP soon after printing and after drying.

**Figure 3:**
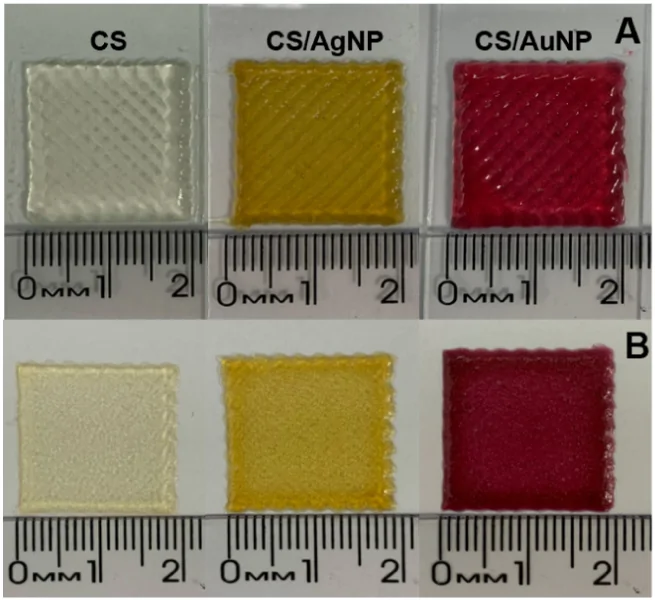
3D-printed CS, CS/AgNP, and CS/AuNP films with size 20 mm (length) × 20 mm (width) × 1 mm (height): (A) soon after printing (wet) and (B) after drying.

The film was printed with dimensions of 20 mm (length) × 20 mm (width) × 1 mm (height). The percentage of printing fidelity was measured after printing (wet), considering the CAD model design. This information is presented in Table 3, along with the average weight.

**Table 3:** Percentage of Printing Fidelity Compared to the CAD Model with Nominal Dimensions of 20 mm × 20 mm × 1 mm (Length × Width × Height) and Mean Weights of 3D-Printed Nanocomposite Films (n = 10)

Sample	Length (%)	Width (%)	Height (%)	Wet weight^a^ (g)	Dry weight^b^(g)	Theoretical metal amount (μmol per film)
CS	96.8 ± 1.6	97.6 ± 1.4	93.5 ± 4.7	0.552 ± 0.042	0.181 ± 0.012	-
CS/AgNP	98.6 ± 2.0	99.0 ± 1.3	102.5 ± 5.4	0.571 ± 0.036	0.186 ± 0.005	0.442 ± 0.028
CS/AuNP	99.4 ± 1.5	99.8 ± 1.0	104.0 ± 5.2	0.597 ± 0.033	0.192 ± 0.007	0.463 ± 0.025

a Soon after printing.

b After drying for 72 h at room temperature.

It is possible to observe fidelity higher than 90% of the 3D-printed films when compared with the CAD model. This can be related to the thixotropic nature of hydrogels, as their ability to recover viscosity contributes to sustain the shape. SSE 3D printing offers high printing fidelity and allows for precise customization of the geometry and dimensions of constructs, as shown in Figure S3. This versatility is a significant advantage of 3D-printed biomedical materials, enabling individualized dose adjustments tailored to each patient’s specific therapeutic needs. Additionally, personalized devices can improve treatment adherence. Customization options range from designing dressings that fit the size and depth of a wound to creating more patient-friendly geometries, all of which can enhance comfort, acceptance, and overall compliance with the treatment.

Regarding the average weights, the films showed a water loss of ∼67% after drying at room temperature. The CS/AgNP and CS/AuNP films exhibited a higher weight in comparison to CS, most likely due to the higher quantity of solids in the formulation. Moreover, the small standard deviation in the dry weight of the CS/AuNPs and CS/AgNP films indicates that the addition of nanoparticles sustains good printing reproducibility. This could be attributed to the homogeneous dispersion of nanoparticles within the chitosan matrix, which prevents nozzle clogging and ensures excellent reproducibility in the printing process, making the hydrogels suitable candidates for the 3D printing of devices in biomedical applications.

The presence of nanoparticles was characterized in the 3D-printed nanocomposite films by acquiring their UV–vis spectra (Figure 4). In both nanocomposite films, the characteristic SPR bands were observed at 417 nm for CS/AgNPs and 533 nm for CS/AuNPs, which confirms the presence of nanoparticles in the films.

**Figure 4:**
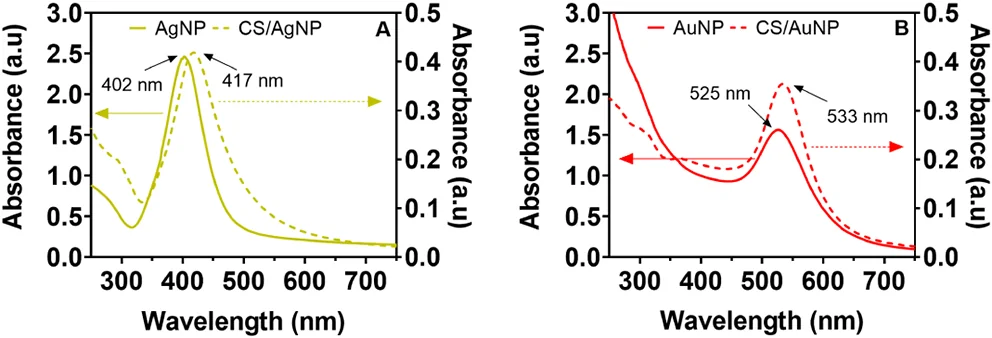
UV–vis spectra of nanoparticles: (A) AgNPs (aqueous dispersion) and CS/AgNP (3D-printed film) and (B) AuNPs (aqueous dispersion) and CS/AuNP (3D-printed film).

Figure 5 shows SEM images of the CS, CS/AgNP, and CS/AuNP films obtained with secondary electron (Figure 5A) and backscattered electron (Figure 5B) signals.

**Figure 5:**
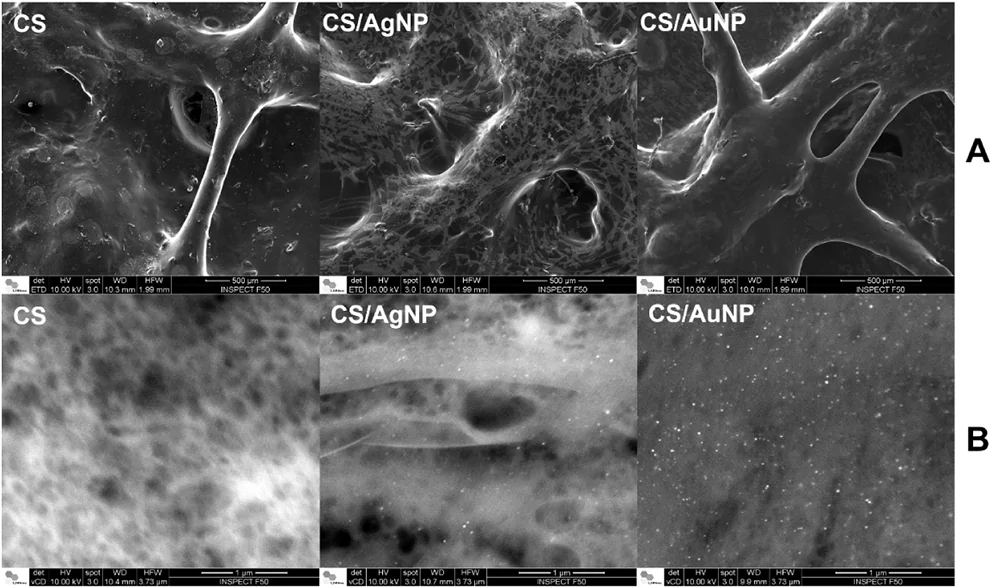
SEM images of CS, CS/AgNP, and CS/AuNP obtained with (A) secondary electron signal and (B) backscattered electron signal.

Figure 5A shows the surface morphology of the films, which demonstrates a network of filaments on a submicrometric level. A better filament definition was observed for CS/AuNPs, which aligns with the rheological properties previously discussed. In Figure 5B, the backscattered electron signal reveals regions with different chemical compositions. The heavier elements in the sample (gold and silver, in this case) backscatter electrons more efficiently than the light ones (organic chains), and then metal nanoparticles are revealed as bright points homogeneously dispersed across the chitosan films in samples CS/AgNP and CS/AuNP. As expected, these bright spots were not observed in the CS films.

Complementing the SEM images, PIXE and *μ*-PIXE analyses were performed to identify gold and silver in the samples and qualitatively assess the distribution of the nanoparticles in the polymeric matrix. Figure S4 shows the *μ*-PIXE maps of silver (Lα and Lβ) and gold (Lα, Lβ, and Mα) in CS/AgNP and CS/AuNP, respectively, acquired over areas of 1 mm^2^, evidencing the presence and broad distribution of the nanoparticles across the sampled areas in the films. This set of analyses enabled the detection of silver and gold within the polymer matrices and confirmed the distribution of the metallic nanoparticles throughout the 3D-printed nanocomposite films. These findings are consistent with the previous results, which indicated homogeneous nanoparticle dispersion in the hydrogel, ensuring a reproducible 3D printing process.

The TGA data of 3D-printed films are presented in Figure S5. Up to 150 °C, the thermograms show a weight loss of 25–27% for CS, CS/AgNP, and CS/AuNP nanocomposites, which was attributed to the water content in the films, demonstrating the film’s capacity to retain water.^57^ Further weight losses correspond to the main decomposition of the organic components (chitosan and glycerol), occurring between 150 and 300 °C. Although this degradation onset is lower than that of raw chitosan, the 3D-printed films, regardless of nanoparticle incorporation, remain thermally stable up to 150 °C.

The behavior in water of the 3D-printed nanocomposite films is shown in Table 4.

**Table 4:** Behavior in Water of 3D-Printed Nanocomposite Films[Table-fn tbl4fn1]

Samples	Swelling (%)	Dry mass loss (%)
CS	156.0 ± 4.4^a^	48.2 ± 5.5^a^
CS/AgNP	54.1 ± 9.2^b^	43.5 ± 4.0^a^
CS/AuNP	53.5 ± 3.1^b^	50.5 ± 2.6^a^

i Values are presented as mean ± SD (n = 3). Different superscript letters indicate statistically significant differences between groups within the same column (*p* < 0.05).

Regarding the swelling in water indices, all of the 3D-printed films swelled in water, as expected for hydrogel-derived chitosan films. A decrease in swelling of CS/AgNP and CS/AuNP films was observed in comparison with CS films. This effect may be attributed to the interactions between the nanoparticles and the polymer matrix.^75,76^ Nanoparticles have been reported to promote crosslinking and/or electrostatic interactions with the functional groups of chitosan, which reduce chain mobility and decrease the free volume that would typically be occupied by water.^77–79^ Recent studies indicate that nanoparticle incorporation can influence the swelling behavior of polymeric nanocomposites, with higher nanoparticle contents often associated with a more constrained polymer network and reduced chain mobility.^80^ Previous studies on chitosan–AgNP systems have associated reduced swelling with interactions of silver species with hydroxyl and amine groups of chitosan, which may restrict chain mobility and water penetration.^81^ In AgNP-containing carboxymethyl starch/poly­(vinyl alcohol) hydrogels, XRD analysis indicated structural changes in the polymeric network after nanoparticle incorporation, which were associated with reduced swelling.^82^ Similarly, AuNP incorporation into polysaccharide hydrogels has been associated with a tighter polymeric structure and reduced leaching of low-molecular-weight components.^83^ Zhang et al. also reported reduced swelling in AgNP- and AuNP-containing poly­(vinyl alcohol) membranes, associated with changes in the polymer network.^84^ Collectively, these findings support the experimental results observed in the nanocomposite films, reinforcing the role of metal nanoparticles in enhancing the structural stability of polymer membranes. Despite the decrease in swelling, the CS/AgNP and CS/AuNP still swelled around 50% of their weight, which is favorable for wound-dressing applications, for controlling excessive wound exudates while keeping a moist environment at the wound site.^85^ On the other hand, all the 3D-printed films presented similar dry mass loss in water, which suggests that nanoparticle incorporation did not substantially affect overall solid-material loss under the tested conditions.

The pH of the skin can vary by region and is generally slightly acidic. This acidity is essential for maintaining the function of the skin barrier, regulating the microbiome, and supporting the healing process. However, when the skin suffers an injury, the pH in that area can change significantly.^86^ The nature of the wound—whether acute or chronic—plays an important role in these changes. In the initial phase of an acute wound, under controlled environments without infections, the pH is typically acidic, below 6.0, due to the activity of neutrophils involved in wound healing.^49,87^ In chronic wounds, the pH is generally more alkaline, ranging from pH 7.15 to 8.9. This alkalinity favors bacterial growth and inflammation and hinders the healing process.^49,50^

Therefore, Figure 6 presents the behavior of 3D-printed nanocomposite films in aqueous environments, monitoring their relative mass at three different pH values pH 4.0, 5.8, and 8.5.

**Figure 6:**
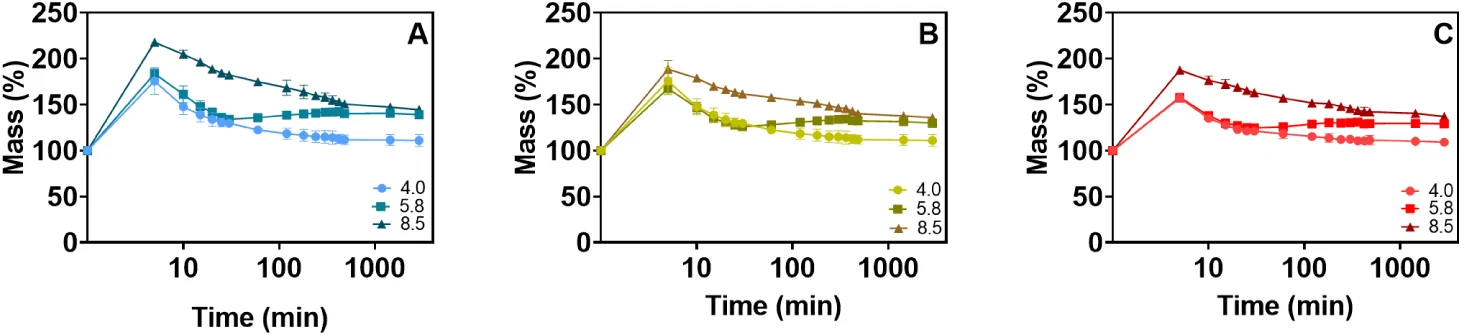
Behavior in an aqueous environment at pH values of 4.0, 5.8, and 8.5 for (A) CS, (B) CS/AgNP, and (C) CS/AuNP (n = 3).

The 3D-printed films presented, in all tested pH values, a two-phase behavior. Initially, a rapid water uptake is observed in the first 5 min, attributed to the high hydrophilicity of the chitosan matrix and the polymer network’s initial openness to solvent diffusion. Following the initial swelling, there was a gradual decrease in the relative mass. Changes in relative wet mass alone cannot distinguish water uptake from actual loss of solid material, since both processes occur simultaneously during immersion. Glycerol is water-soluble, and the 3D-printed films contain approximately 30% glycerol; therefore, its leaching during immersion may contribute to the observed mass changes. To further distinguish hydration from material loss, dry mass loss was determined after 24 h of exposure to the same pH conditions, and the results are presented in Table 5. All 3D-printed films exhibited dry mass loss at the three tested pH values, indicating the loss of solid components from the matrices. This may be due to the leaching of water-soluble components, such as glycerol, and/or partial solubilization of loosely bound polymer chains. At pH 8.5, a trend of lower mass loss was observed than at pH 5.8 and 4.0 for all films. Chitosan is soluble in acidic conditions due to the ionization state of the amino groups, therefore, at pH 4.0 and 5.8, the solubilization is faster. At an alkaline pH of 8.5, the amino groups are largely deprotonated, reducing chitosan solubility and favoring retention of the polymeric matrix.

**Table 5:** Dry Mass Loss of 3D-Printed Nanocomposite Films in Buffered Media at Different pH (n = 4)[Table-fn tbl5fn1]

	Dry mass loss (%)		
Samples	pH 4.0	pH 5.8	pH 8.5
CS	20.8 ± 2.5^ab^	23.7 ± 4.3^a^	14.4 ± 2.7^b^
CS/AgNP	22.4 ± 5.1^a^	21.6 ± 3.1^a^	12.0 ± 1.4^b^
CS/AuNP	21.4 ± 1.1^a^	26.0 ± 1.2^b^	11.3 ± 3.0^c^

i Values are presented as mean ± SD (n = 4). Different superscript letters indicate statistically significant differences between groups within the same row (*p* < 0.05).

The addition of nanofillers can significantly impact the mechanical properties of polymer matrices. This is primarily due to the high specific surface area and nanoscale dimensions of the particles, which affect the interfacial interactions within the composite.^88^ As observed in Figure 7, the incorporation of nanoparticles into a chitosan matrix resulted in a reduction in tensile strength (TS) and an increase in the extensibility (E) of chitosan films. This behavior can be attributed to the interference of nanoparticles in the intermolecular interactions between the chitosan chains, which can lead to a polymeric network that is less rigid and more flexible.^89^ Other studies demonstrated a similar behavior regarding the incorporation of silver nanoparticles into the chitosan matrix, observing a decrease in TS and an increase in E of the films, a phenomenon directly related to the concentration of nanoparticles used.^75^

**Figure 7:**
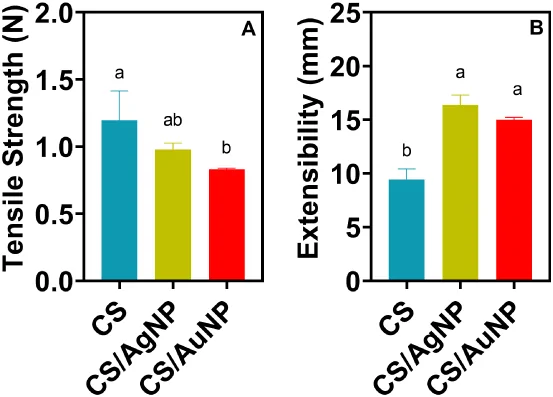
Tensile strength (A) and extensibility (B) of 3D-printed films: CS, CS/AgNP, and CS/AuNP (n = 3). Different superscript letters indicate statistically significant differences between groups (*p* < 0.05).

### 3.4 Biological Characterization

*Ex vivo* assays for bioadhesion (Figure 8) showed that the maximum force of adhesion (adhesiveness) did not show statistically significant differences between the groups. However, the films containing AuNPs (CS/AuNP) exhibited higher values for both the work of adhesion and the debonding distance. This behavior indicates that incorporating nanoparticles can enhance energy dissipation during the displacement process without increasing the peak force.^90^ This effect may be linked to the enhanced extensibility or viscoelasticity of the polymer matrix induced by the presence of nanoparticles. Even if the maximum force value remains unchanged, the film requires more total energy to be detached completely. This leads to a higher work of adhesion and a longer debonding distance. These findings are consistent with the results obtained from the rheological and mechanical assays, which suggest relevant structural modifications in the matrix following nanoparticle incorporation.

**Figure 8:**
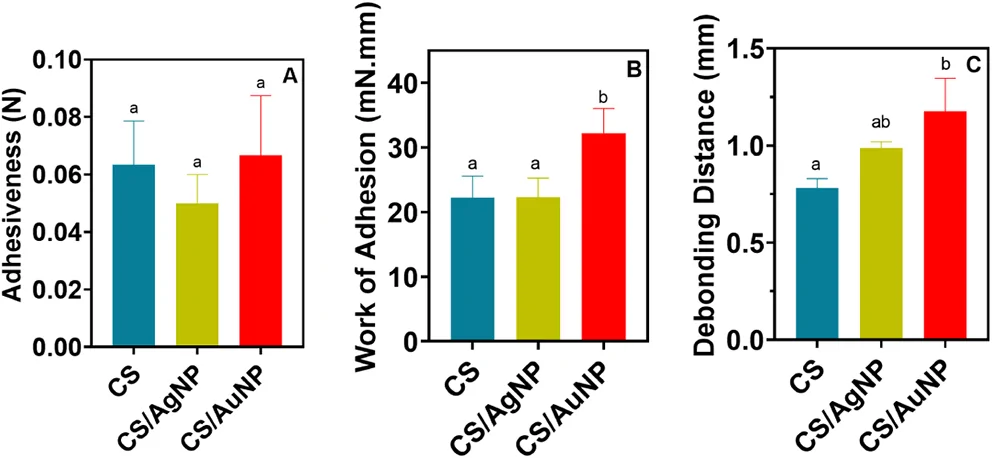
Adhesiveness (A), work of adhesion (B), and debonding distance (C) of 3D-printed films: CS, CS/AgNP, and CS/AuNP (n = 3). Different superscript letters indicate statistically significant differences between groups (p < 0.05).

The antimicrobial activity of CS, CS/AgNP, and CS/AuNP was evaluated with *Pseudomonas aeruginosa*, *Staphylococcus aureus*, and *Candida albicans*, and the results are shown in Figure 9.

**Figure 9:**
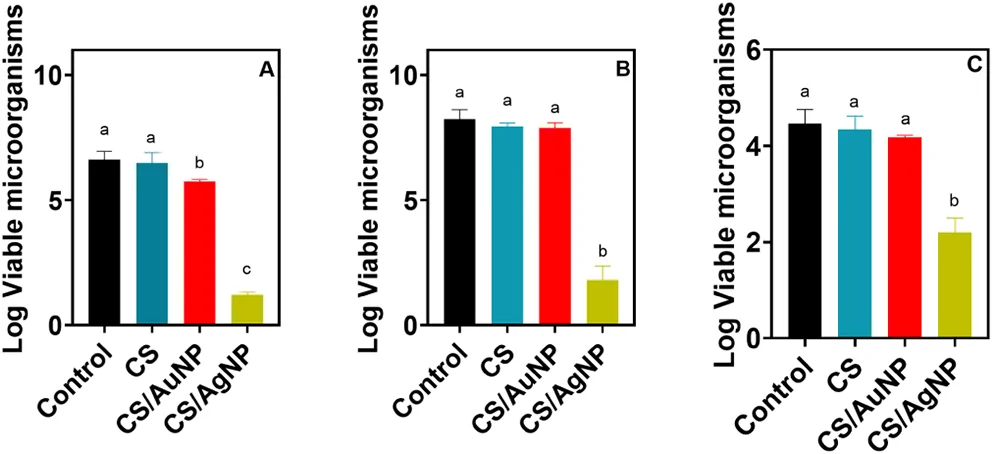
Antimicrobial activity of CS, CS/AgNP, and CS/AuNP films against (A) *Staphylococcus aureus*, (B) *Pseudomonas aeruginosa*, and (C) *Candida albicans*. Different superscript letters indicate statistically significant differences between groups (*p* < 0.05).

CS/AgNP and CS/AuNP demonstrated greater antibacterial than antifungal activity against the tested microorganisms; this may be due to the higher microbial resistance and greater cellular complexity of eukaryotic organisms such as fungi, particularly *Candida albicans*.^91–93^ Despite both films containing nanoparticles of the same shape and size, the CS/AgNP film demonstrates greater antimicrobial activity compared to the CS/AuNP film. In fact, the antimicrobial activity of the CS/AuNP film did not show a significant difference when compared to the control. This result can be attributed to the nature of nanoparticles; the greater potential for ion release of AgNP (Ag^+^) may explain their increased antimicrobial activity. It is important to note that studies have shown both AgNPs and AuNPs with sizes ranging from 10 to 15 nm exhibit significant antimicrobial activity against various microorganisms.^94–96^ This suggests that the size of the nanoparticles plays a crucial role in their biological interactions.^96^ However, the effectiveness of AuNPs as antimicrobial agents is highly dependent on their concentration, as they typically require higher amounts to achieve comparable antimicrobial effects.^95^ Considering the concentration of 0.45 μmol of Au per film used in the present study, previous research has demonstrated that chitosan films containing AuNPs can exhibit antimicrobial activity when higher concentrations of gold are incorporated into the polymer matrix, while still maintaining the stability and dispersion of the nanoparticles.^95^ Amongst many mechanisms that explain the antimicrobial activity of metal nanoparticles, ion release can generate free radicals, causing rupture of the cell membrane and inhibiting DNA replication by interacting with phosphate or sulfur groups.^97^ Although CS/AuNP did not exhibit high antimicrobial activity, studies have demonstrated that chitosan and AuNPs are widely recognized as bacteriostatic or bactericidal.^98^ Further studies could systematically investigate the effects of nanoparticle loading on antimicrobial performance using more concentrated AgNP and AuNP dispersions, while also reassessing stability, hydrogel rheology, and printability. The combined incorporation of AgNPs and AuNPs could also be explored as a strategy to modulate antimicrobial activity.

Moreover, the nanoparticles are trapped within the polymer matrix. In this state, the antimicrobial effect mainly relies on the direct contact between bacteria and the nanoparticles and/or ions present on the film surface.^99,100^ Although this feature plays a key role in antimicrobial action,^99^ on the other hand, the nonliberation of nanoparticles is a positive outcome, considering the safety of films for topical use.

The penetration of the nanoparticles in skin was evaluated using Franz diffusion cells, and the skin samples were analyzed by SEM-EDS to investigate the presence of nanoparticles in the skin layers. Figure 10A and 10D shows the cross-sectional images of the stratum corneum for samples treated with AgNPs and AuNPs (aqueous dispersions, free nanoparticles), respectively. Using the backscattered electron signal, bright spots in Figure 10B and 10E indicated the presence of metal nanoparticles in the skin treated with AgNPs and AuNPs. The nanoparticles were identified through the EDS spectra presented in Figure 10C and 10F, where characteristic peaks of silver and gold were detected in the bright spots. Specifically, the Lα (2.98 keV) and Lβ (3.15 keV) emission lines of silver and the Mα (2.12 keV) and Lα (9.71 keV) lines of gold confirmed their presence at the stratum corneum, indicating interaction with the skin matrix.^101,102^

**Figure 10:**
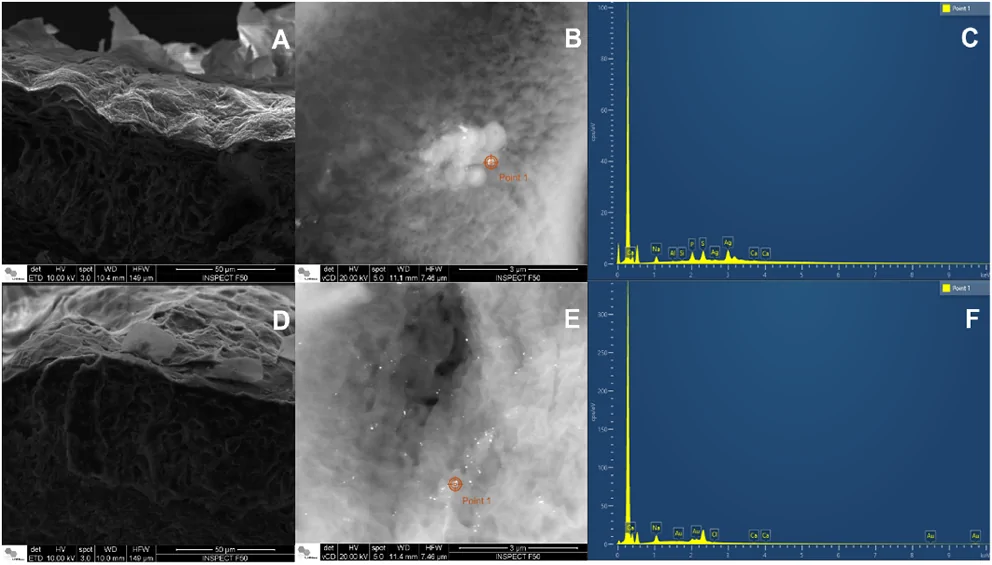
SEM images of skin treated with AgNPs and AuNPs. (A,D) Secondary electron signal images and (B,E) backscattered electron signal images. (C,F) Punctual EDS spectra.

Despite adhering to the stratum corneum, the nanoparticles did not penetrate the skin into its innermost layers. All layers of the skin were analyzed, stratum corneum and deeper layers, to assess the penetration of AgNPs and AuNPs, which were not identified.

Skin was also treated with the 3D-printed films, CS, CS/AgNP, and CS/AuNP, where the nanoparticles are trapped in the polymer matrix. SEM images of the stratum corneum of these skin samples are presented in Figure S6. Unlike the samples treated with AgNP and AuNP dispersions, no nanoparticles were observed in either the upper or the deeper layers of the skin. The absence of nanoparticles in the skin treated with the 3D-printed nanocomposite films suggests that they remained trapped within the films, indicating a potentially safer topical application. Future studies should incorporate appropriate biocompatibility assessments, damaged skin models, physiologically relevant release media, and in vitro wound infection models to more accurately replicate the complex physiological conditions of the wound environments.

## 4 Conclusions

In this study, AuNPs and AgNPs were successfully synthesized at the nanometric scale, with average sizes ranging from 9 to 12 nm. The nanoparticles were effectively incorporated into chitosan-based hydrogels, which demonstrated favorable rheological properties, including viscosity and thixotropic behavior, making them compatible with 3D printing by using the SSE technique. The hydrogel formulations exhibited pH values above 4, which falls within the recommended range for topical skin applications. The printed films demonstrated printing fidelity exceeding 90% when compared to the CAD model along with good reproducibility, as indicated by the average mass of the films. Spectroscopic and imaging analyses confirmed the uniform distribution of nanoparticles throughout the polymeric matrix. Antimicrobial assays showed a more significant activity for CS/AgNP films. *Ex vivo* porcine skin studies indicated that unlike free nanoparticles, metals were not detected in skin samples treated with the 3D-printed films, supporting effective nanoparticle confinement within the chitosan matrix and limited skin penetration under the tested conditions. Overall, the developed 3D-printed nanocomposite films show strong potential as personalized topical antimicrobial materials, combining high print fidelity with physicochemical, structural, and biological attributes that are relevant to biomedical applications.

## Supplementary Material



## Notes

UV–vis spectra and TEM images of AuNPs and AgNPs; photographs of 3D-printed films in customized sizes; *μ*-PIXE maps and thermogravimetric curves of CS/AuNP and CS/AgNP 3D-printed films; and SEM images of porcine skin treated with CS, CS/AgNP, and CS/AuNP 3D-printed films (PDF)
