# Supplementary material for: Development of Antimicrobial Chitosan-Silver and Chitosan-Gold Nanocomposite Films by 3D Printing for Skin Application
Source: ACS Omega. 2026 Sep 11;11(37):56346–59. doi: 10.1021/acsomega.6c07342 (PMC13613387; doi:10.1021/acsomega.6c07342)
Supplement: Supplementary file 1 [file ao6c07342_si_001.pdf]

Supporting Information

Development of Antimicrobial Chitosan-Silver and Chitosan-Gold Nanocomposite Films by 3D Printing for Skin Application

Rita de Cássia dos Reis Schmidt[a], *Vitória Espinosa de Oliveira*[b], *Tainá Griebler*[b], *Júlia Lazzarini Machado*[a], *Leonardo Pessoa da Silva*[c], *Henrique Trombini*[b], *Marcelo Barbalho Pereira*[c], *Cesar Liberato Petzhold*[d], *Gabrielle Aguiar Dantas*[e], *Roberto Christ Vianna Santos*[e], *Nadine Lysyk Funk*[f], *Ruy Carlos Ruver Beck*[f], *Karina Paese*[f], *Monique Deon*[a]*

[a] Universidade Federal de Ciências da Saúde de Porto Alegre, Programa de Pós-Graduação em Biociências, Rua Sarmento Leite 245, CEP 90050-170, Porto Alegre, RS, Brazil;

[b] Universidade Federal de Ciências da Saúde de Porto Alegre, Rua Sarmento Leite 245, CEP 90050-170, Porto Alegre, RS, Brazil;

[c] Universidade Federal do Rio Grande do Sul, Instituto de Física, CP 15051, CEP 91501-970, Porto Alegre, RS, Brazil;

[d] Universidade Federal do Rio Grande do Sul, Instituto de Química, CP 15003, CEP 91501-970, Porto Alegre, RS, Brazil;

[e] Laboratory of Oral Microbiology Research, Universidade Federal de Santa Maria, Av. Roraima 1000, CEP 97105-900, Santa Maria, RS, Brazil.

[f] Universidade Federal do Rio Grande do Sul, Programa de Pós-Graduação em Ciências Farmacêuticas, Av. Ipiranga 2752, CEP 90610-000, Porto Alegre, RS, Brazil.

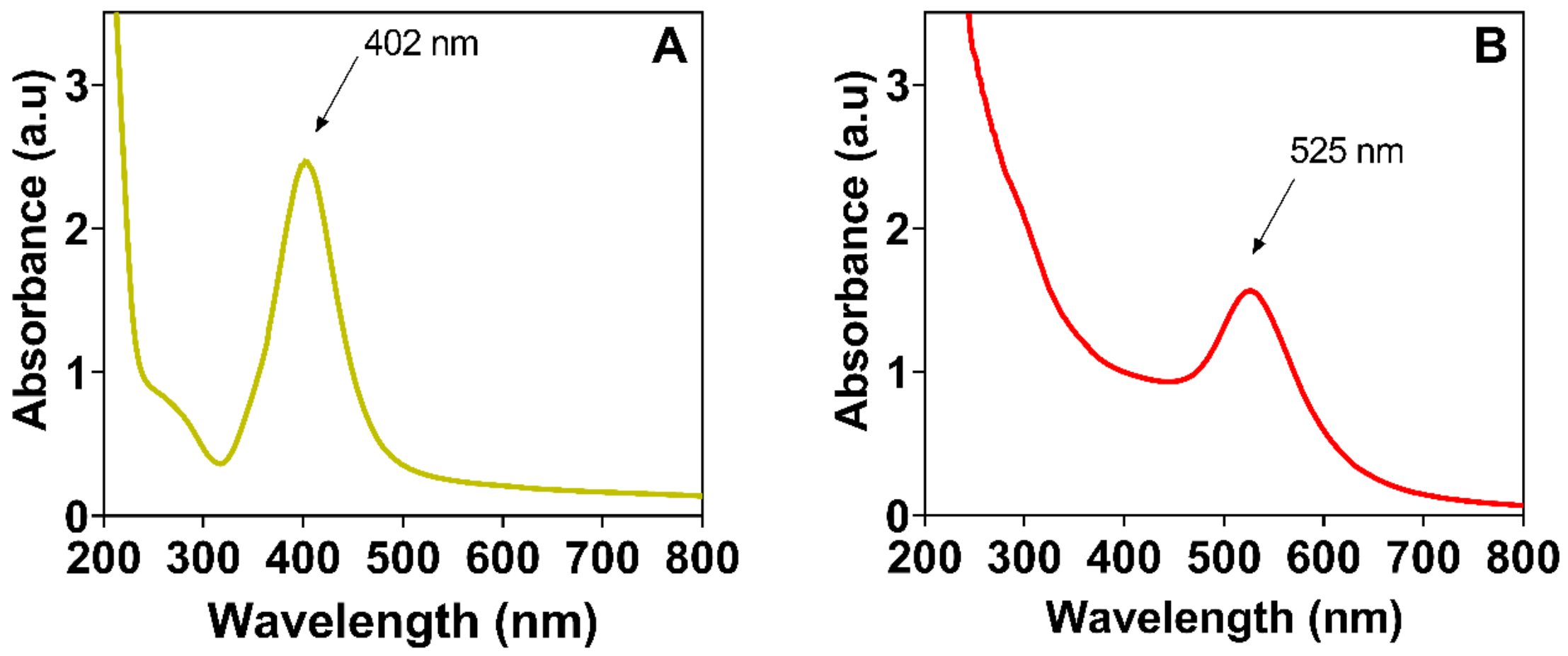

Figure S1. UV-Vis spectra of (A) AgNP and (B) AuNP aqueous dispersions.

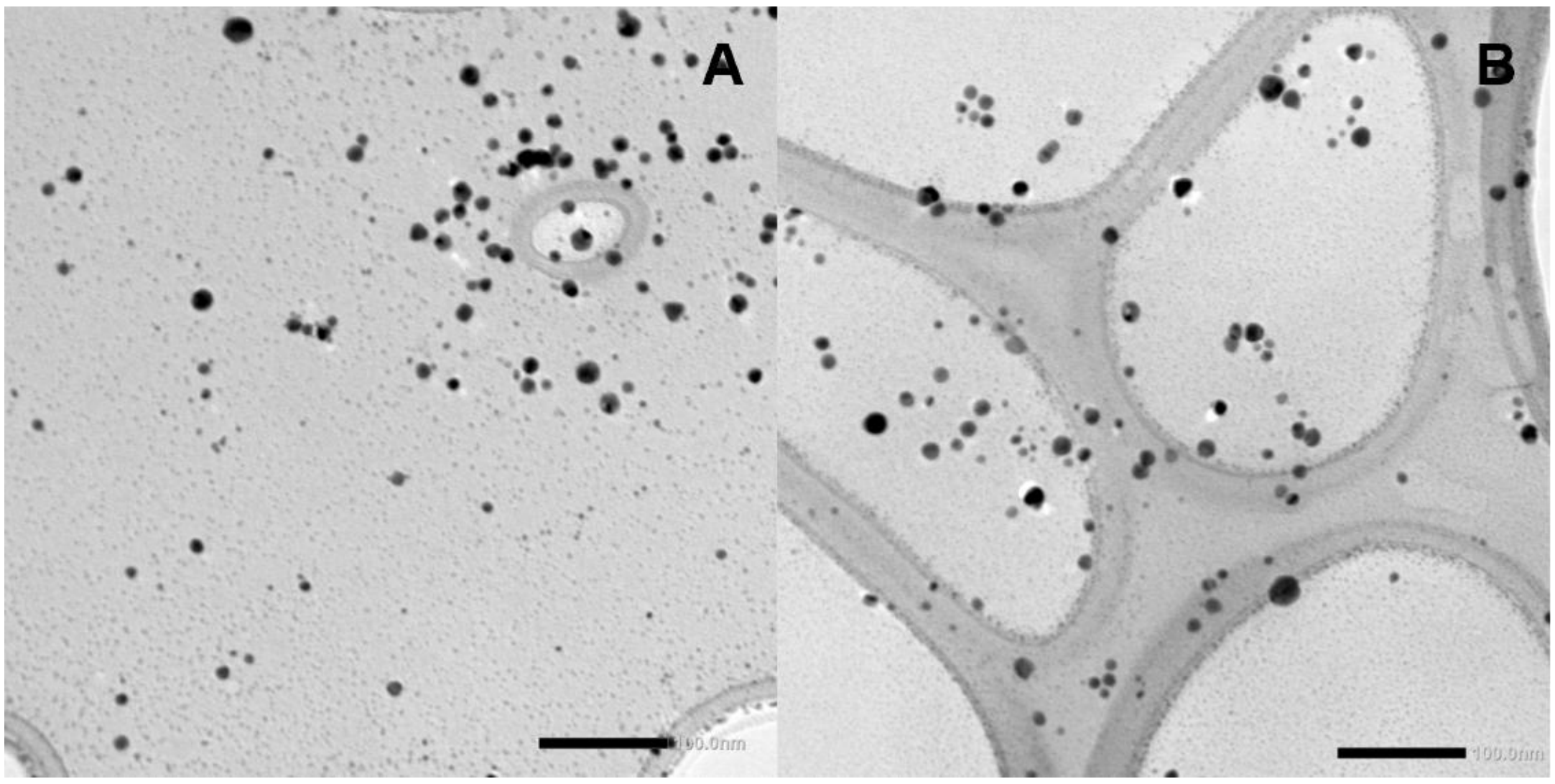

Figure S2. TEM images of (A) AgNP and (B) AuNP.

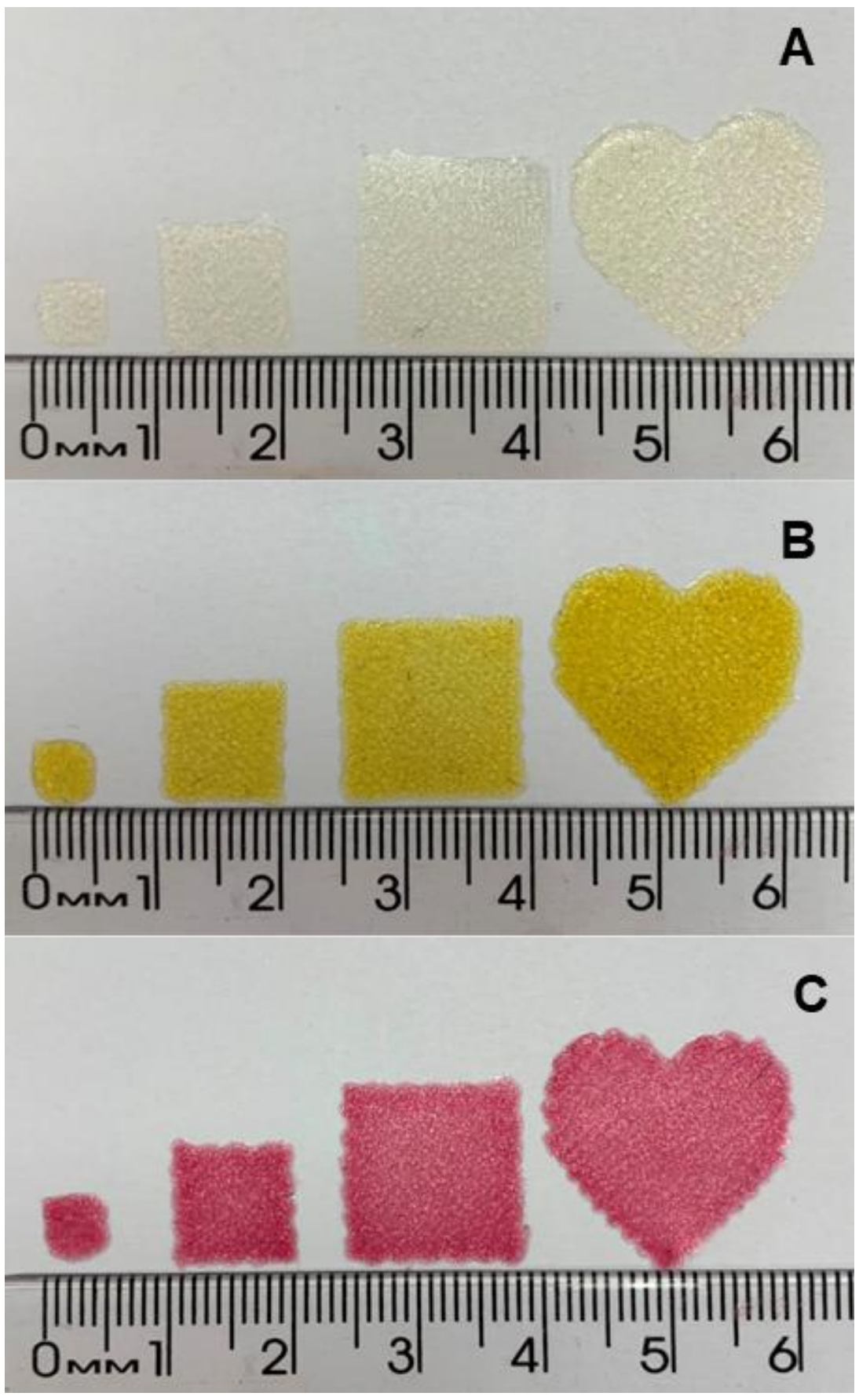

Figure S3. SSE 3D-printed films customized in different sizes and shapes (A) CS, (B) CS/AgNP and (C) CS/AuNP.

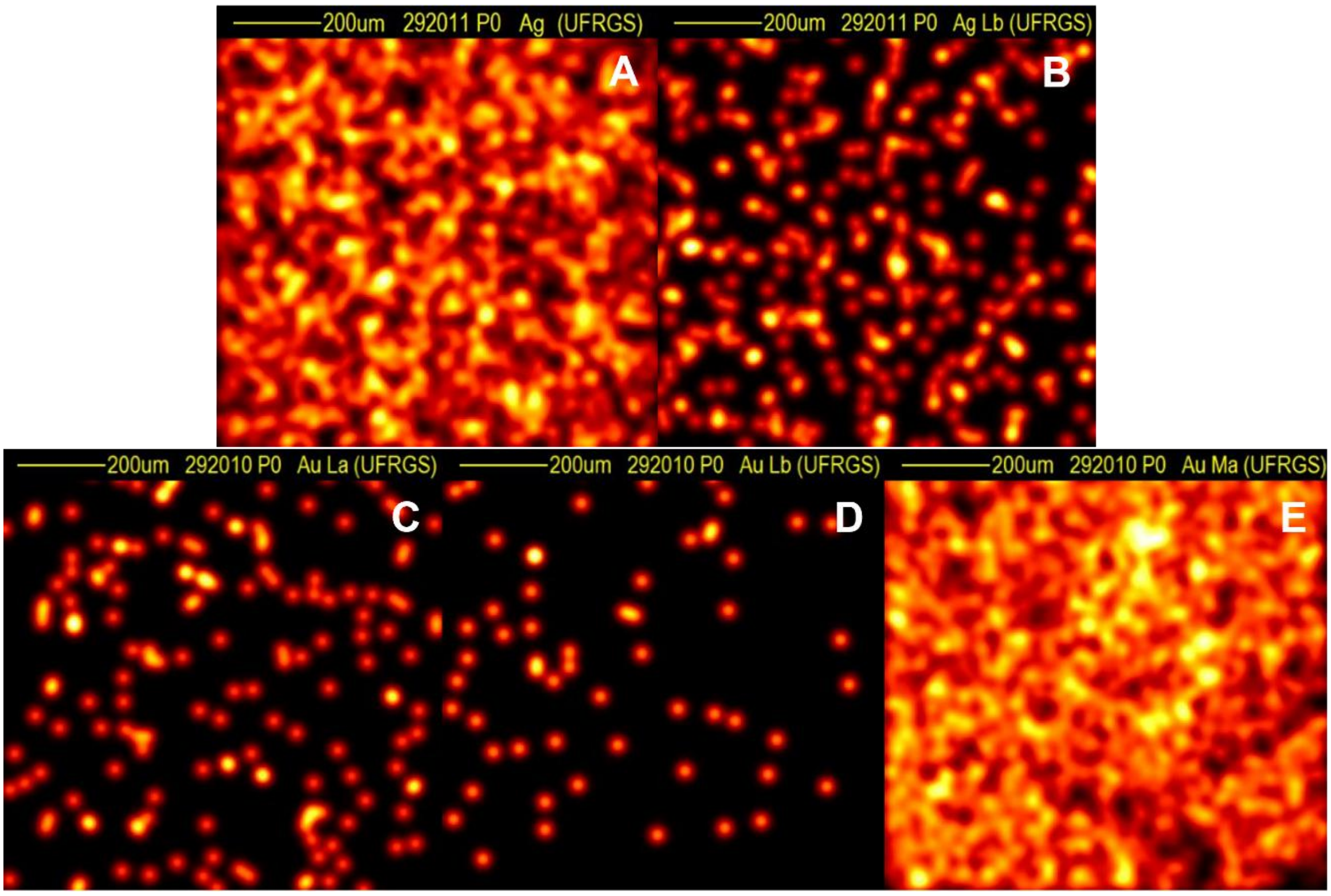

Figure S4. μ-PIXE maps of (A) Lα line of Ag in CS/AgNP, (B) Lβ line of Ag in CS/AgNP, (C) Lα line of Au in CS/AuNP, (D) Lβ line of Au in CS/AuNP, and (E) Mα line of Au in CS/AuNP. Scanning area of 1 mm^2.

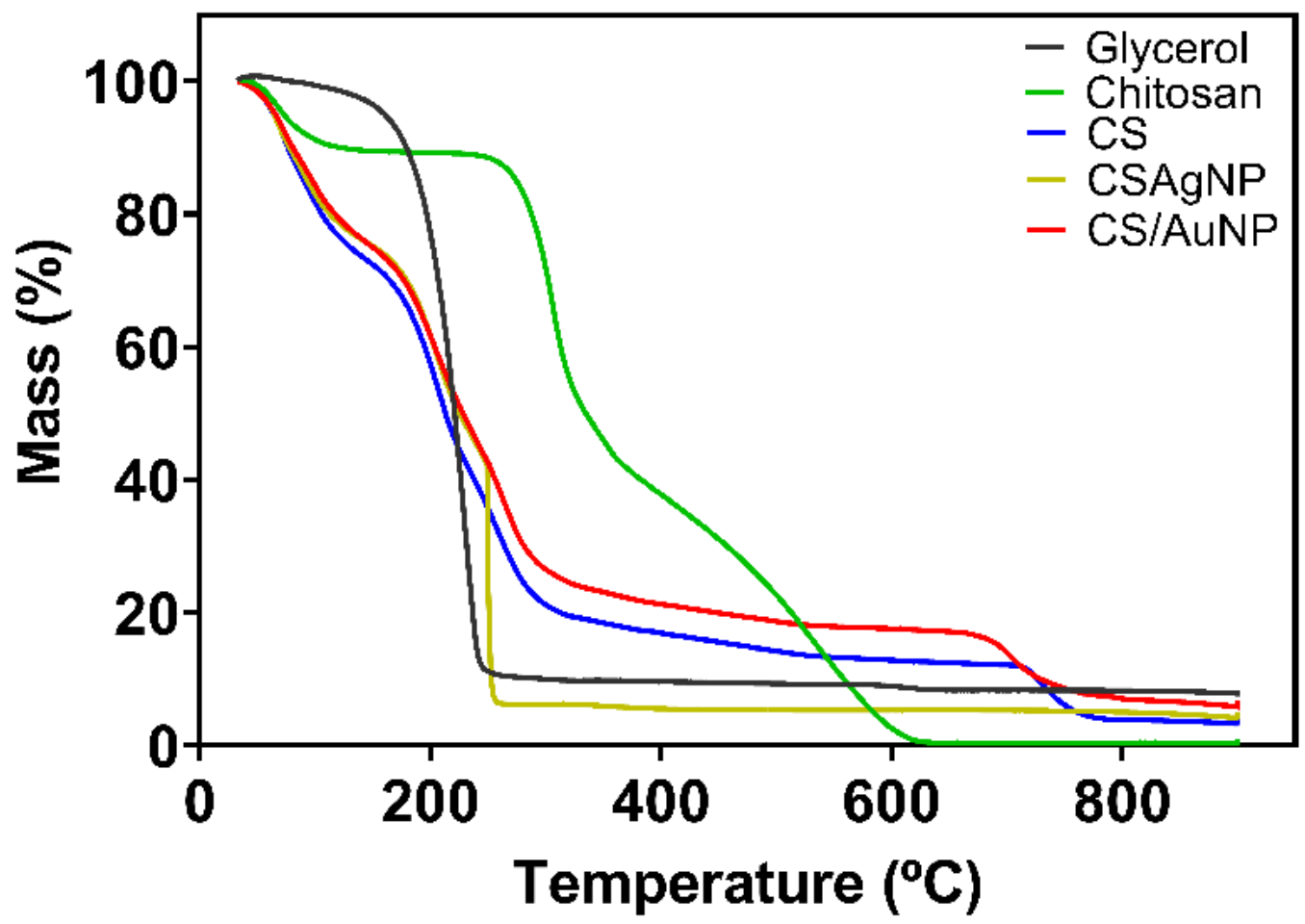

Figure S5. Thermograms of CS, CS/AgNP, CS/AuNP films, chitosan raw material and glycerol.

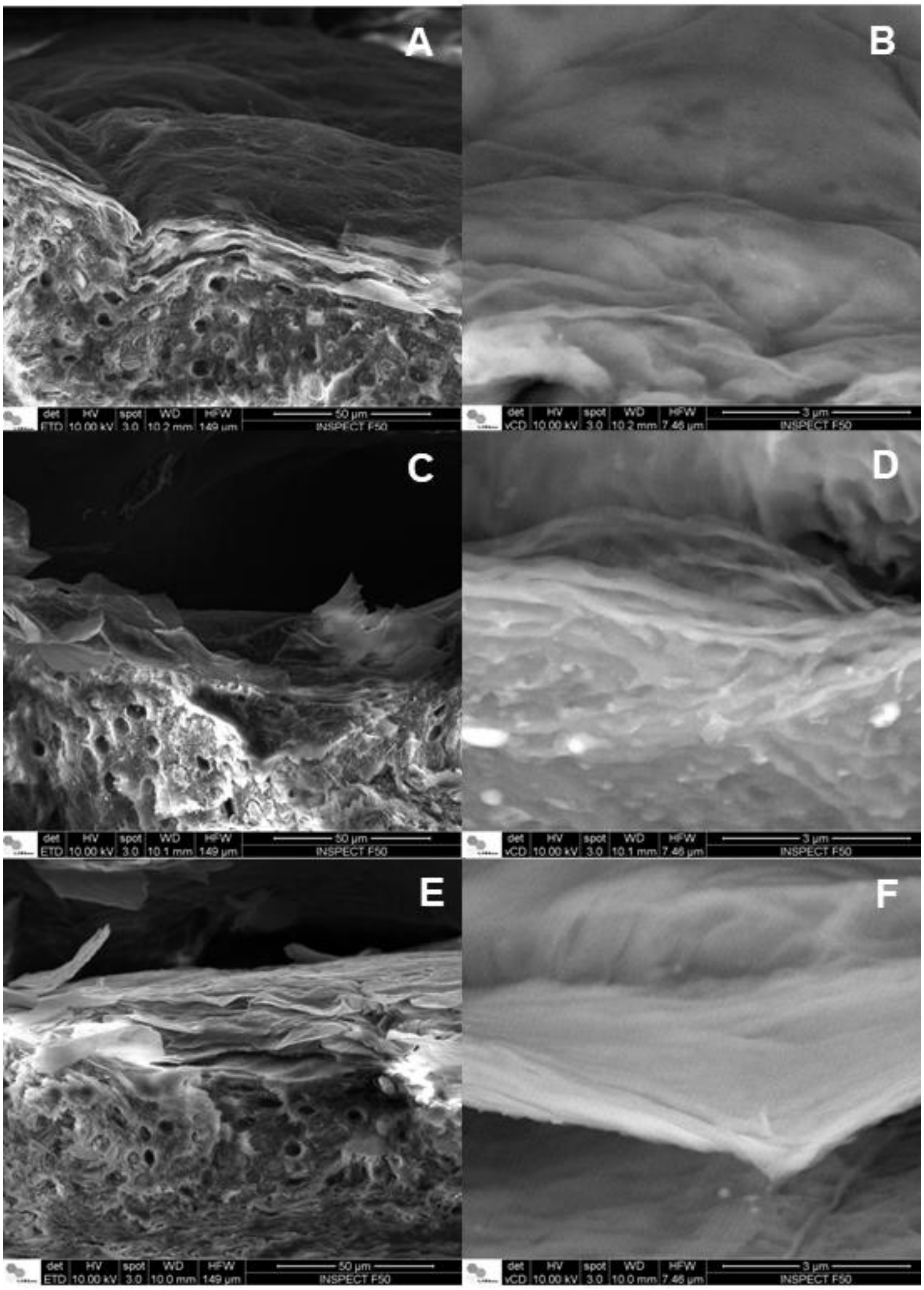

Figure S6. SEM images of skin treated with (A, B) CS, (C, D) CS/AgNP, and (E, F) CS/AuNP. (A, C and E) Secondary electrons signal images (B, D and F) backscattered electrons signal images.
